# Online toolkits for collaborative and inclusive global research in urban evolutionary ecology

**DOI:** 10.1002/ece3.11633

**Published:** 2024-06-25

**Authors:** Amy M. Savage, Meredith J. Willmott, Pablo Moreno‐García, Zuzanna Jagiello, Daijiang Li, Anna Malesis, Lindsay S. Miles, Cristian Román‐Palacios, David Salazar‐Valenzuela, Brian C. Verrelli, Kristin M. Winchell, Marina Alberti, Santiago Bonilla‐Bedoya, Elizabeth Carlen, Cleo Falvey, Lauren Johnson, Ella Martin, Hanna Kuzyo, John Marzluff, Jason Munshi‐South, Megan Phifer‐Rixey, Ignacy Stadnicki, Marta Szulkin, Yuyu Zhou, Kiyoko M. Gotanda

**Affiliations:** ^1^ Department of Biology & Center for Computational and Integrative Biology Rutgers University – Camden Camden New Jersey USA; ^2^ Department of Biological Sciences, Center for Computation & Technology Louisiana State University Baton Rouge Louisiana USA; ^3^ Institute of Evolutionary Biology, Faculty of Biology, Biological and Chemical Research Centre University of Warsaw Warsaw Poland; ^4^ Department of Urban Design and Planning University of Washington Seattle Washington USA; ^5^ Virginia Polytechnic and State University Entomology Department Blacksburg Virginia USA; ^6^ School of Information University of Arizona Tucson Arizona USA; ^7^ Centro de Investigación de la Biodiversidad y Cambio Climático & Facultad de Ciencias de Medio Ambiente Universidad Indoamérica Quito Ecuador; ^8^ Center for Biological Data Science Virginia Commonwealth University Richmond Virginia USA; ^9^ Biology Department New York University New York New York USA; ^10^ Research Center for Territory and Sustainable Habitat Universidad Indoamérica Quito Ecuador; ^11^ Department of Biology Washington University of St. Louis St. Louis Missouri USA; ^12^ Ecology and Evolutionary Biology University of Toronto Toronto Ontario Canada; ^13^ Frankfurt Zoological Society Frankfurt Germany; ^14^ Louis Calder Center & Department of Biological Sciences Fordham University Armonk New York USA; ^15^ Department of Biology Drexel University Philadelphia Pennsylvania USA; ^16^ Department of Geological and Atmospheric Sciences Iowa State University Ames Iowa USA; ^17^ Department of Biological Sciences Brock University St. Catharines Ontario Canada

**Keywords:** collaborations, decolonization, eco‐evolutionary dynamics, international, science communication, urban ecology, urban evolutionary ecology

## Abstract

Urban evolutionary ecology is inherently interdisciplinary. Moreover, it is a field with global significance. However, bringing researchers and resources together across fields and countries is challenging. Therefore, an online collaborative research hub, where common methods and best practices are shared among scientists from diverse geographic, ethnic, and career backgrounds would make research focused on urban evolutionary ecology more inclusive. Here, we describe a freely available online research hub for toolkits that facilitate global research in urban evolutionary ecology. We provide rationales and descriptions of toolkits for: (1) decolonizing urban evolutionary ecology; (2) identifying and fostering international collaborative partnerships; (3) common methods and freely‐available datasets for trait mapping across cities; (4) common methods and freely‐available datasets for cross‐city evolutionary ecology experiments; and (5) best practices and freely available resources for public outreach and communication of research findings in urban evolutionary ecology. We outline how the toolkits can be accessed, archived, and modified over time in order to sustain long‐term global research that will advance our understanding of urban evolutionary ecology.

## Graphical abstract

Advancing urban evolutionary ecology will require a global perspective, yet many barriers currently prevent scholars from the global south and north from effectively collaborating. We developed toolkits that are flexible, reliable, and accessible on a website to facilitate effective collaborations across geographic boundaries in urban evolutionary ecology.

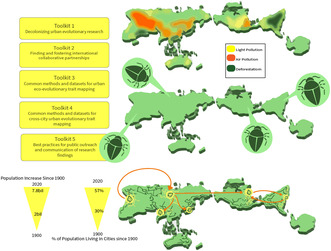

## 1 INTRODUCTION

Urban ecosystems are increasingly being recognized by ecologists and evolutionary biologists as hotbeds for comparative and experimental studies of contemporary evolutionary ecology (Alberti, 2015; Alberti et al., 2017; Rivkin et al., 2019; Szulkin et al., 2020). These studies could potentially distinguish the new and complex selection pressures found in urban landscapes from those that are influential in nonurban systems. The field of urban evolutionary ecology has great potential for interdisciplinary research. Fundamental questions from diverse perspectives in evolutionary ecology have recently been addressed in light of urbanization, including trait plasticity (Campbell‐Staton et al., 2020; Diamond, Chick, Perez, Strickler, & Martin, 2018; Diamond, Chick, Perez, Strickler, & Zhao, 2018); evolutionary parallelism (Santangelo et al., 2022; Winchell et al., 2023); morphological, physiological, and behavioral adaptations (Carlen et al., 2021; Gotanda, 2020; Harris & Munshi‐South, 2017; Miranda, 2017; Winchell et al., 2016); and how ecosystem type (e.g., geographic, habitat type, socio‐economics, etc.) might influence gene flow and genetic diversity (Miles et al., 2019; Noël et al., 2007; Schmidt & Garroway, 2022; Unfried et al., 2013; Wilson et al., 2011).

While prior studies on evolutionary ecology have evaluated the effects of urbanization on ecological and evolutionary dynamics separately, researchers are now beginning to explore these dynamics in the context of eco‐evolutionary dynamics, whereby ecological processes drive evolutionary changes, which then lead to reciprocal effects of evolutionary changes on ecological processes (Hendry, 2017). Such eco‐evolutionary dynamics might play out differently in urban areas due to unique combinations of ecological stressors and both adaptive and neutral mechanisms of evolution in cities (Alberti, 2015; Alberti et al., 2017; Rivkin et al., 2019). Growing interest in studies of urban eco‐evolutionary dynamics is expanding the breadth of research in this sub‐field (Verrelli et al., 2022). Research advances include the integration of other disciplines, such as those that examine sociological, cultural, and political factors and investigate the impacts of racism across urbanizing landscapes (Des Roches et al., 2021; Schell et al., 2020). Given the global scope of urbanization, collaborations among researchers from different world regions will facilitate a greater understanding of the key environmental drivers, traits, and ecological dynamics that underlie evolutionary change in cities.

Advancing knowledge about urban evolutionary ecology will require global perspectives, international collaborations, and cross‐city research. Urban areas are often called “natural laboratories,” but the extent to which global cities are replicates of the stressors and evolutionary and ecological responses that result from urbanization is not yet clear (Santangelo et al., 2022; Verrelli et al., 2022). Cross‐city approaches include taxa that inhabit cities characteristic of both similar and different sociocultural, political, economic, and ethnically diverse populations, which also provide unique opportunities to determine how these factors drive urban eco‐evolutionary dynamics (Alberti et al., 2020; Des Roches et al., 2021; Schell et al., 2020; Schmidt & Garroway, 2022). Moreover, big data technologies are becoming increasingly available and affordable worldwide, making it possible to generate large comparative databases within and across many geographically distinct cities. Thus, we are poised to advance knowledge about urban evolutionary ecology at a global scale and to make robust predictions about how populations, communities, and entire ecosystems respond to anthropogenic change drivers across urbanizing landscapes.

Currently, a substantial geographic bias exists in both framing and conducting urban evolutionary ecology research, with most of the studies receiving widespread attention originating in the Global North (e.g., first‐world countries north of the equator; Aronson et al., 2017; Christie et al., 2021; Martin et al., 2012; Pyšek et al., 2008; Shackleton et al., 2021). As is true for science more generally, much of this bias results from legacies of racism (Schell et al., 2020), historic barriers to the inclusion of nonwhite scholars in scientific research (Lee, 2020), and the practice of under‐or‐devaluing nonwestern traditions of knowledge generation (Miriti et al., 2023). Some research practices of Western researchers who conduct studies in foreign countries, such as the practices of Western scientists conducting research in other countries without including local collaborators or distributing scientific contributions inequitably (“Parachute Science”; Miller et al., 2023; Stefanoudis et al., 2021), and telling researchers from other regions how to conduct their studies (Strambach & Surmeier, 2018) exacerbate these disparities. If new global collaborative studies are built without consideration of this historic context and local scientists, they will perpetuate these inequities. Thus, we must advance science in general and urban evolutionary ecology in particular by following a path that is more collaborative and fundamentally decolonial. We agree that the labor of facilitating a transition to more equitable systems of data acquisition and management should be borne by scholars who have historically benefited from historical inequities (i.e. Western researchers). However, advancing knowledge in urban evolutionary ecology will require systems that not only include contributions from scholars from the Global South but also give them proper credit for their contributions (Nuñez et al., 2021; Pettoreli et al., 2021).

The Global North and Western homogenization of data collection, management, sharing, and collaborating limits its accessibility to scientists in different contexts. At the same time, standardized protocols, data management, and communication are needed to allow researchers to better leverage their data in order to advance knowledge about urban evolutionary ecology through large‐scale studies (Barney et al., 2015; Borer et al., 2014). We argue that increased resources for international collaboration, a common online space for researchers to find freely available research infrastructure (e.g. databases, maps, protocols, etc.), opportunities for the co‐creation of content that gives proper credit to contributors, and increased appreciation for multiple modes of knowledge generation could help scholars to navigate this tension and, ultimately, advance our understanding of evolutionary ecology across urbanizing landscapes.

**FIGURE 1:**
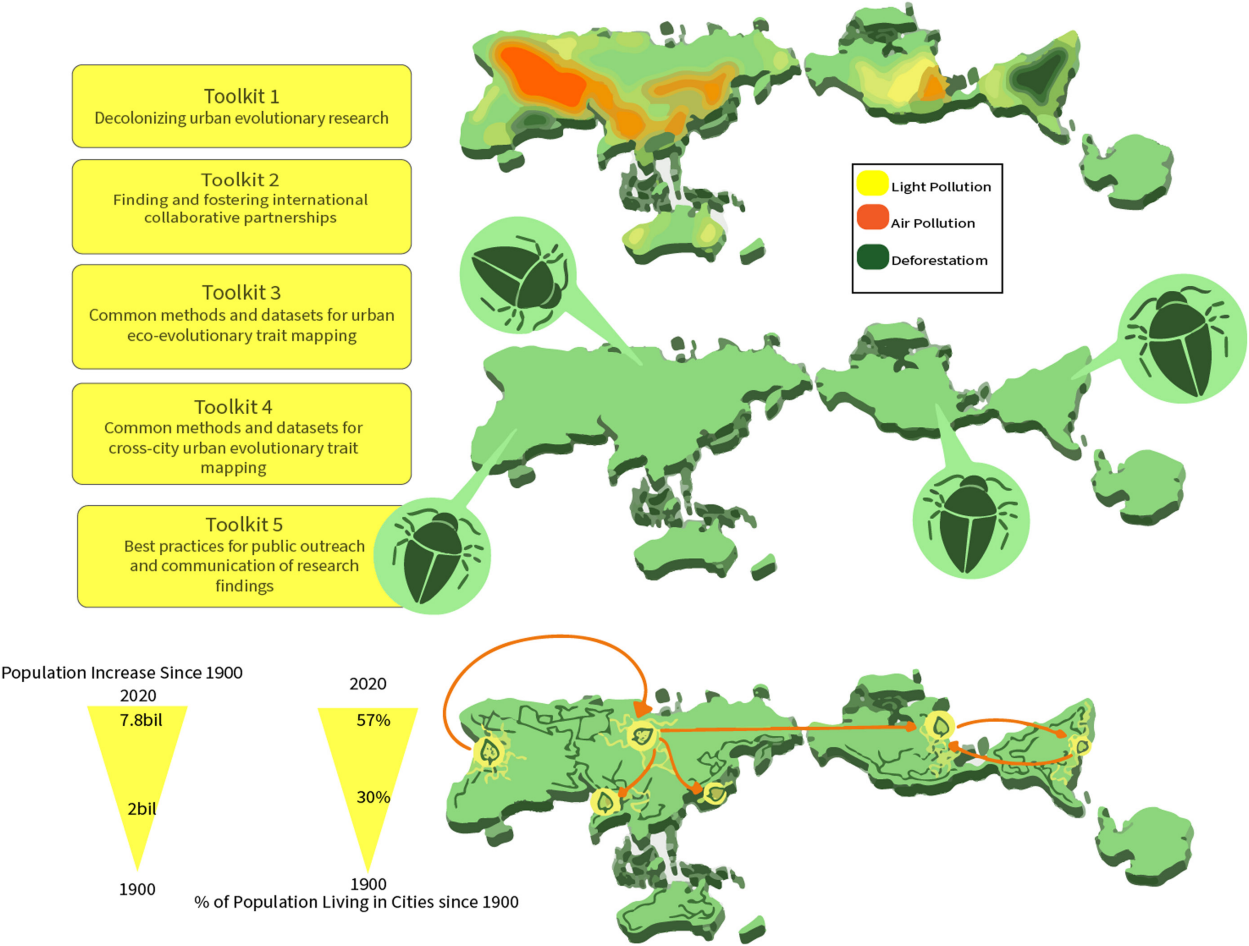
All 5 toolkits represented interacting with an AuthaGraph map projection. Each tool targets a different challenge in the global study of urban evolution. The top map represents global areas of light pollution, deforestation, and heat stress. The middle map displays common species found across the globe, which could benefit from a globally collaborative perspective. The third map displays impervious ground cover through the representation of major highways. Green leaves represent species and traits distributed in different cities, arrows circulating between them display global connectivity, and the potential ability to amass global trait‐mapping datasets. On the bottom left, triangles represent both the human population increase and the proportion of the human population living in cities. In an effort to decolonize this research, the projection used to create the figure is the Authagraph map projection, which decentralizes areas of colonial power (e.g. Europe, USA).

Technological advances have led to the generation of multiple freely available tools that can help urban evolutionary ecology researchers advance global knowledge in an equitable fashion. However, these resources are haphazardly distributed across the internet, with many resources not often specifically earmarked as “urban,” and thus, often require prior knowledge about the proper search terms to be useful. At this pivotal time in the emergence of the field of urban evolutionary ecology, we need an online “one‐stop shop” that promotes equitable international collaborations and includes access to these freely available resources. We developed a website that serves as this online collaborative research hub for those interested in urban evolutionary ecology research. We envision this website as a portal that serves people from all geographic, cultural, ethnic, and professional backgrounds who are interested in urban evolutionary ecology. The goals of this article are twofold: (1) we present a perspective on five key barriers to effective, collaborative, international research in urban evolutionary ecology, and (2) as part of the discussion of each barrier, we describe the section of our new website that focuses on addressing that specific barrier. These discussions and online resources are organized into five toolkits that span the lifetime of the project and provide a decolonizing approach to facilitating greater global collaborations in urban evolutionary ecology research (Figure 1). In order to maintain its utility over time, the website will be regularly updated and archived, with the flexibility to include new content as it becomes available. Here, we describe the motivation, methods, initial content for this online space, and our vision for growing the site as a space for co‐creation.

## 2 METHODS

### 2.1 Description of the initial content on the website

The Urban Evolutionary Ecology tools website (Figure 1; https://www.urbanevoecotools.org/index.html) will initially have nine pages: a home page with an interactive and co‐created map, a general “about” page, a page to acknowledge content contributors, a contact us page, and individual pages for each toolkit in relation to urban evolutionary ecology research: (i) decolonizing urban eco‐evo research, (ii) finding and fostering international collaborative partnerships, (iii) common methods and datasets for urban trait mapping, (iv) common methods for cross‐city trait mapping, and (v) best practices for public outreach and communication. Each page is expanded upon in the appropriate section below. The website also includes opportunities to contribute to the toolkits and an interactive map. The website was built using Weebly, an eCommerce company that is owned by Square, and the domain name was purchased through GoDaddy.

### 2.2 Accessibility and contributions

This website is freely available to anyone who has internet access. We have designed it to be streamlined, minimizing the need for extensive bandwidth or data usage. We used DeepL (https://www.deepl.com/) for the initial translation of the website content into Spanish and then had individuals who are fluent in Spanish review the translations. We plan to, in addition, translate the website to include the 10 most common languages spoken in the world (Beytía et al., 2022): English, Mandarin Chinese, Hindi, Spanish, French, standard Arabic, Bengali, Russian, Portuguese, and Indonesian.

This website is a living resource that will continue to expand and adapt to enhance international collaborations on urban evolutionary ecological research. To accomplish this goal, we have developed forms that will enable individuals to contribute and expand the toolkits (Appendices [Link], [Link]). Individuals are asked which toolkit they are contributing to (including an option for additional toolkits), what the contribution is, and their contact information. Contact information will be made available on the website only if the individual grants permission. We invite researchers outside of the current list of contributors to contribute resources and dialog about this resource.

### 2.3 Methods for mapping and community contributions

**FIGURE 2:**
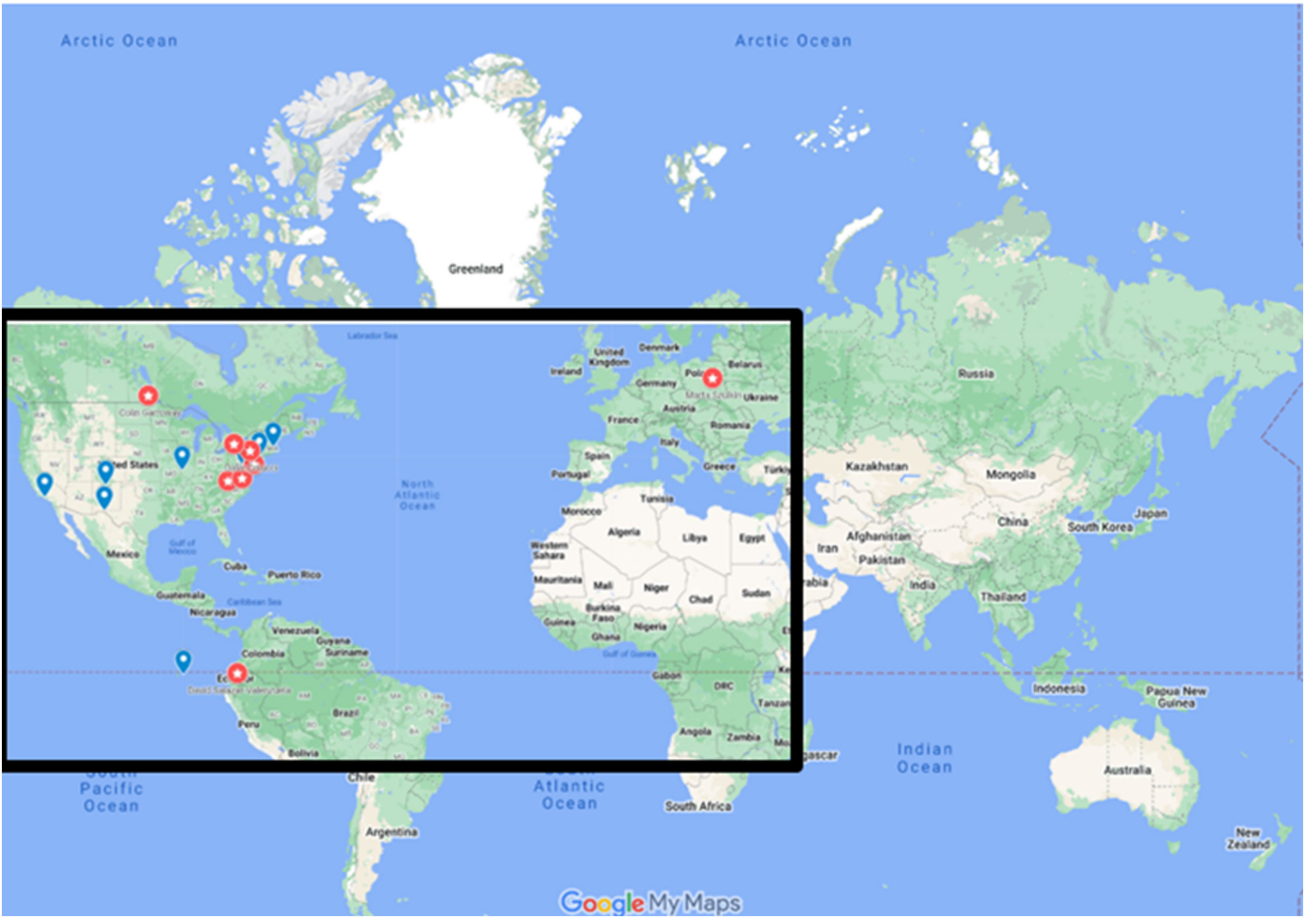
Screenshot of the current version of the MURP (Map of urban research & projects). The map contains three layers: (1) locations of researchers studying urban ecology and evolution (blue symbols), (2) locations of research projects in urban ecology and evolution (red symbols), and (3) locations of science communicators (yellow symbols). All points on the map are populated through user responses to Google Forms. As the project grows, we hope to increase the number of data points across the map, but especially in the Global South.

The website includes an interactive map called MURP (Mapping Urban Researchers and Projects; Figure 2) that crosses multiple toolkits and is co‐created by users. MURP is a Google Map that is populated with data independently written by contributors in the fields of urban ecology and science communication. This site has the potential to grow and invite researchers from a variety of disciplines to participate in this field of knowledge. Specifically, we developed simple Google Forms for (1) researchers in the field of urban evolutionary ecology, (2) urban evolutionary ecology research projects, and (3) science communicators (Appendices [Link], [Link]). These forms ask users to fill in the decimal latitude and longitude of their locations (institutes, field sites, cities, etc.), so they can be visually represented on the map. The map will be regularly updated.

### 2.4 Updating and archiving

We will update the website and the MURP using data from submitted Google forms, with periodical quality checks. We will regularly archive html and pdf versions of the website and CSV versions of the data from our forms (Appendices [Link], [Link]) on Github (https://github.com/photopidge/Urban‐Evolutionary‐Ecology‐Toolkit) to maintain the public accessibility of toolkit information over time.

## 3 TOOLKITS

### 3.1 Decolonizing urban evolutionary ecology research

In urban systems, it is particularly important to recognize that colonialism and imperialism were major drivers in the development of urban industrial capitalism and the formation of many cities. From 1500 to 1950 nearly every region in the world's economy was at one time controlled by a European core power (King, 1990). Even with re‐independence and new global cities, a need remains to understand contemporary urbanism by investigating its colonial past. Similarly, the development of sciences, including ecology and evolution, has been shaped by historical colonialism.

In the interest of more just science, we must acknowledge how ongoing colonial contexts influence the funding, creation, implications, and dissemination of scientific thought at both local and global scales (Miriti et al., 2023). Globally, we need to address how we fund science, how we conduct work in areas in which we do not live, and how to equitably and respectfully collect and disseminate information. Locally, we need to address how we conduct research in a respectful and equitable manner, how we can actively engage local communities throughout the entire research process, and how a researcher's positionality influences the way in which they ask questions and analyze data (Baker et al., 2019). In other words, we need to explicitly work toward decolonizing science.

Decolonizing science is the process of explicitly addressing and actively working against the disproportionate legacy of the dominance of Western culture on research and science education and the ways that science is biased by Western thought. Because of this history, science has benefited from and been used to falsely justify Western colonialism and cultural superiority. Furthermore, ecologists and evolutionary biologists have a responsibility to understand the history of our field and how our research is shaped by colonial thought. For example, European ecologists contributed to the domination of colonized lands using ecological concepts to discount Indigenous knowledge systems and justify the exertion of environmental and social control (Baker et al., 2019; Trisos et al., 2021). At the same time, European scientists developed foundational ideas and concepts through the study of these regions (Baker et al., 2019). This racist and colonial history has shaped the fundamental values, terminology, and concepts of ecology and evolution and continues to influence those who study ecological and evolutionary dynamics to this day (Cheng et al., 2023; Miriti et al., 2023).

The paradigms that the ecology and evolution of nonhumans can be understood outside of social context and that human societies can be understood without considering their eco‐evolutionary context have recently been challenged (Mackenzie & Jeggo, 2019; Trisos et al., 2021). Moreover, imposing Western research priorities and methods onto local communities in other regions infringes upon local values and knowledge systems, generates uneven power dynamics, and further concentrates the power of those who already have it (Nadasdy, 1999). Furthermore, the propensity for Western scientists to disregard different kinds of knowledge systems, such as traditional ecological knowledge, blocks the potential for increased understanding and functional impact on the systems we study. Addressing these concerns is particularly important in urban systems since many cities around the world were built on Indigenous land, especially those in regions such as the Americas, Australia, and New Zealand, where European colonization occurred. Unfortunately, this history is often not part of the scientific framing or study timescale in studies of urban evolutionary ecology.

Thus, a paradigm shift is necessary in the ways that we conduct ecological and evolutionary research (Trisos et al., 2021). Studies and databases should value and include a wider range of information formats and efforts to standardize data should recognize that Western methods are not the most appropriate method of understanding for all communities (Nadasdy, 1999). Furthermore, ownership and sovereignty must be a key consideration in the processes of collecting, storing, and sharing data. While Western‐trained scientists often see knowledge as belonging to those who generated it, many Indigenous communities see tribal knowledge as an inherent part of the group's identity and wealth, which even then, might only be held by select members (Harding et al., 2012). As such, western scientists working with people from other cultures must respect their data sovereignty by respecting local ownership and the right to refuse the collection or sharing of information (Trisos et al., 2021). This includes working with Indigenous communities that have been displaced from cities due to colonization. It also includes a recognition that the labor of decolonizing our science needs to be borne primarily by researchers from backgrounds that have historically benefited from colonial systems. While we need to center the experiences of scholars from groups historically and systemically excluded from STEM fields, we need to do so in an equitable manner in which the bulk of the effort is not expended by people from these groups. However, there are multiple systemic barriers to achieving these goals in our current research infrastructure. For example, access to data and scholarly literature remains inequitably distributed, with data extracted from the Global South and hosted in the Global North (Trisos et al., 2021). When possible, promptly uploading data to public archives would increase accessibility for researchers at under‐resourced institutions and allow comparisons of ecological and evolutionary phenomena around the globe.

Here, we provide three tools to help urban ecologists and evolutionary biologists conduct research utilizing a decolonization framework. All of the tools in this toolkit will evolve with independent submissions to incorporate people from different backgrounds and/or those affected by different intersectionalities into our efforts to decolonize urban evolutionary ecology research.

1. *A list of publications on the topic of decolonizing ecology and evolution*: these manuscripts discuss a variety of topics related to the decolonization of ecology and evolution, including; how to work with, recognize, and incorporate non‐Western groups and ways of knowing; anti‐racist, decolonial, and ethical interventions for multiple academic contexts; the importance of researcher positionality; discussion of and guidelines for finding and replacing harmful or problematic names and terminology; the history of the fields; and data ownership, hosting, and access. A number of the included articles deal specifically with decolonization in the digital realm, ranging from descriptions of access and ownership problems to recommendations for potential solutions. This toolkit will help researchers consider and address issues surrounding equitable access to and the inclusion of diverse knowledge sources in online databases.
2. *A list of projects that form a partnership between the local community and scientists*: these projects serve as examples of how researchers can form connections with the local groups, as well as use those connections to co‐create new knowledge with a positive impact on the community. When possible, this list will be hyperlinked to the MURP.
3. *Examples of land acknowledgements, positionality statements, and methods sections from empirical studies that discuss research in the context of the specific regional land history*: these examples, paired with links to relevant background information, provide a jumping off point for scientists to employ tools for decolonization directly into their research. For example, researchers in the St. Louis, MO, USA, urban systems are accumulating documentaries, books, and podcasts that they and others can use to educate themselves on the history of the region.

### 3.2 Finding and fostering international collaborative partnerships for urban evolutionary ecology research

Historically, logistic challenges often caused scientists to focus on a single city or region when studying urban evolutionary ecology. However, researchers who are geographically distant can now connect and work together to facilitate more powerful research outcomes. Identifying colleagues at different global institutions is increasingly important for understanding the similar and different patterns associated with broadly distributed taxa, traits, and processes across different cities and bioregions. This strategy is one of a “division of labor,” where rather than individual laboratories competing for resources and publications, sample and data collection, and analyses, they can be split up among different colleagues depending on how each individual/group can best contribute. Collaborative networks can keep up with emerging methods and technologies and co‐develop standards for methods and data collection that ensure that global scientific studies are accessible, while maintaining communication in reducing redundancies and uncertainties in data collection.

International collaborations have been increasing since the emergence of the Internet in the 1990s (Olechnicka et al., 2019; National Science Board [NSB], 2020; Marginson, 2022), yet global collaborations in ecology and evolution still face numerous barriers (e.g., Smith et al., 2023) that have hindered the advancement of interdisciplinary research in urban ecology and evolution (Verrelli et al., 2022; Westman & Broto, 2022). For example, access to research and scientific resources has been a major issue, as researchers from low‐income countries often lack the necessary funds to access paid journals or attend international conferences (Petersen, 2021). Moreover, language can be a barrier, as English is often the dominant language in scientific communication (Gordin, 2015) and can limit participation from non‐English speaking regions (Ramírez‐Castañeda, 2020; Steigerwald et al., 2022; Tardy, 2004). Immigration policies and visa restrictions have created additional obstacles for researchers trying to collaborate across borders (Casanova et al., 2020; Feeney et al., 2023; Trost et al., 2018), particularly in countries where science and academia are seen as potential threats to national security (Cohen & Marquardt, 2019; Lee & Haupt, 2020). Furthermore, parachute science (see introduction; Miller et al., 2023; Stefanoudis et al., 2021), has also been shown to represent a significant barrier to global collaborations (Asase et al., 2022; Genda et al., 2022). Overcoming these and other associated barriers (e.g., discrimination based on different racial, cultural, gender, sexual orientation, ability status, or religious identities) will require a concerted effort to address issues of equity, accessibility, and cultural sensitivity in the global scientific community (Cheng et al., 2023; Cheruvelil et al., 2014; de Grijs, 2015; Ferrini‐Mundy, 2013; Harding et al., 2012; Ramírez‐Castañeda et al., 2022).

Starting international partnerships can be challenging when researchers are not aware of one another's work until they see a published article, a problem that is only exacerbated by systemic inequities. Once researchers find international partners, their collaborations in urban evolutionary ecology can be facilitated using a variety of tools that support communication, project management, and data sharing across borders. However, the appropriate combination of tools that facilitate collaboration in particular topics, contexts, and times might vary and may be difficult to define. Here, we develop a basic toolkit that aims to increase the ease in starting and continuing international collaborations. This toolkit will initially have three sets of tools:

1. *Collaborator finder tools*: We developed a map of urban evolutionary ecologists and the urban‐related projects they are working on. We also developed user‐friendly Google forms that will allow anyone who accesses the site to add their information. This map can help facilitate connecting researchers across the globe with each other and with potential resources. Language barriers can be overcome by either allocating funds to hire translators before, during, or after the collaboration process or using support tools such as DeepL to ensure that researchers communicate effectively among themselves and that findings are accessible to a wider audience (Steigerwald et al., 2022). We will initially translate our web‐based interface into the most commonly used global languages, with opportunities to increase this capability through future funding efforts.
2. *Online repository of freely available tools to facilitate collaborative international research*: Academic databases and social networks, such as Scopus, Web of Science, ResearchGate, ResearcherID, and even Twitter, can help researchers identify potential collaborators and build lasting partnerships between researchers. Communication tools, such as Zoom, Skype, Slack, WhatsApp, Discord, and Microsoft Teams can help researchers communicate effectively across time zones and international borders. Managing meetings across different time zones can be facilitated by tools like World Time Buddy, while project management can be streamlined using platforms like Miro, Trello, Notion, or even by creating projects in GitHub. Our toolkit will serve as a repository for these tools, with the functionality for users to suggest more tools to include on the website.
3. *Online repository of freely available tools for analyzing and publishing data from international collaborative research*: Collaborative writing platforms like Google Drive, Overleaf, and HackMD can be used to co‐author papers, while code management platforms like GitHub can be used to manage and collaborate on software projects. Data sharing can be facilitated using cloud storage services like Dropbox, the MEGA, or Box cloud storage services, while reference management and discovery can be supported by platforms like Zotero, Citavi, and Mendeley. We also recommend considering using project management tools such as Open Science Framework (OSF), which integrates many of the tools listed above (e.g. GitHub, GoogleDrive).

### 3.3 Common methods and freely available datasets for urban eco‐evolutionary trait mapping

Functional traits—physiological, morphological, or phenological characteristics of species (Violle et al., 2007)—can draw direct connections between ecological or evolutionary observations and their underlying processes and inform potential outcomes (Grime, 1974; Jeliazkov et al., 2020; Zakharova et al., 2019). Functional traits can also serve as a common currency to allow direct comparisons across space, time, and biological taxa (Zakharova et al., 2019), and increase the resulting sample size by sampling effort. Trait‐based research thus has been widely used to explain biodiversity patterns, assess ecological functions and ecosystem services, and provide decision‐makers with early‐warning signals (Jeliazkov et al., 2020; Zakharova et al., 2019). Over the past decades, we have accumulated many measurements of functional traits for the most common taxonomic groups to form global trait databases (Gallagher et al., 2019). However, their applicability to urban studies is limited, as data from existing global species trait databases were largely collected from natural systems. Moreover, many species only have a small number of measures and little information about intraspecies variability. Yet, urbanization can generate novel environmental conditions that can force species to have different, sometimes even extreme, values for important functional traits compared to trait values in natural conditions (Borowy & Swan, 2020; Grilo et al., 2022; Johnson et al., 2018). To better understand the evolutionary ecological consequences of urbanization, urban‐associated trait databases are required, and acquiring such data in urban areas poses its own set of challenges (Santos et al., 2023; Winchell et al., 2022). Such databases can be used to more accurately evaluate the urban filtering of individuals and species (Fournier et al., 2020), urban evolution and adaptation (Thompson et al., 2016), urban ecosystem functions and services (Grilo et al., 2022), and urban degradation and resilience (Morelli et al., 2020).

Most published global datasets of species traits (e.g., TRY, BIEN, AVONET) are based largely on measurements from natural ecosystems or mixtures of natural and anthropogenic systems. As far as we know, no coordinated global urban‐associated trait datasets exist, though some datasets such as SPI‐Birds do have information on whether the data were collected in urban or nonurban areas (Culina et al., 2021). At a local to regional scale, a few such datasets are available, including National Science Foundation (NSF) urban Long Term Ecological Research (LTER) sites (USA; Grimm et al., 2000), PhenObs (mostly Europe; Nordt et al., 2021), as well as those from local studies (Buchholz et al., 2020; Pataki et al., 2013; Philpott et al., 2019; Santangelo et al., 2022; Teixeira et al., 2022). While the methods for gathering trait data are similar across natural and urban sites, urban sites pose additional challenges (Dyson et al., 2019; Thompson et al., 2016; Winchell et al., 2022). Nonetheless, these nonurban trait data will provide critical context to urban studies.

Urban sites are highly variable at very fine spatial and temporal scales, due to the synergistic effects of urban microclimate, pollution, land fragmentation and degradation, and socio‐economic conditions and human activities (Buchholz et al., 2020; Johnson & Munshi‐South, 2017; Thompson et al., 2016), characteristics that have significant consequences for species traits (Borowy & Swan, 2020; Grilo et al., 2022; Johnson et al., 2018). Controlling for urban heterogeneity might not always be possible or desirable, since sampling in urban areas could involve complex administrative processes or dangerous situations (Dyson et al., 2019; Winchell et al., 2022). When possible, researchers should document the local conditions or sampling location, so that other collaborators or research teams can access relevant biophysical information (Rega‐Brodsky et al., 2022). Environmental conditions and species assemblages can be more homogeneous across urban areas than between urban areas and their surroundings (McKinney, 2006). Collaborative projects have capitalized on such homogeneity across urban areas to study global patterns for urban species traits (e.g., Aronson et al., 2016; Santangelo et al., 2022). However, collaborative efforts are still uncommon (see international collaborations and experiment toolkits), with multiple researchers studying the same traits independently across cities (Knapp et al., 2021). Building global urban‐associated species trait databases requires global collaborations based on principles of transparency, mutual respect, and equitable input and acknowledgment (Rega‐Brodsky et al., 2022). In addition, collaborations can benefit from involving local and/or municipal authorities, NGOs, and community groups, which may already collect species traits and could use the research outcomes to benefit local communities.

**FIGURE 3:**
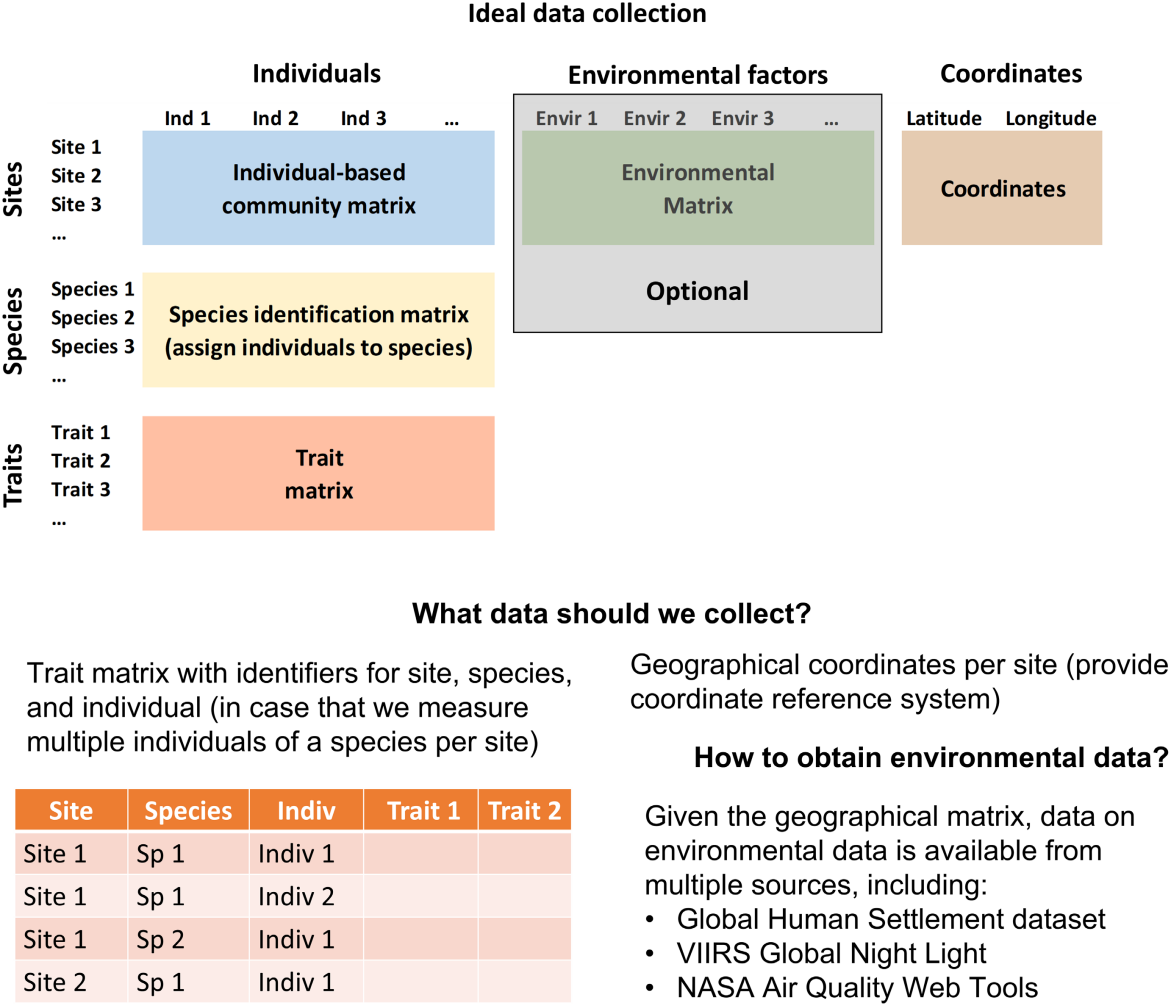
Content of appropriate trait databases. This diagram depicts the kinds of trait data that will be facilitated by the toolkit focused on trait data. The left panel shows the integration of environmental, geographic, trait, and taxonomic data that should be included in global trait databases (Jeliazkov et al., 2020) and the ideal‐case scenario with individual‐specific trait data per location. The right panel shows an example of the data collection table, and sources to obtain environmental data post‐collection (by the same or different researchers).

Current trait databases can be queried by geographic location to obtain trait data at urban locations. In addition, site coordinates can be used to obtain values for multiple urbanization characteristics and stressors, including population density and built area, heat island effects, pollution, and socio‐economic indicators (Brezzi et al., 2012; Falchi et al., 2016; Florczyk et al., 2019; Huang et al., 2019; Mennitt & Fristrup, 2016). However, geographic data is not available for all trait sources, and environmental data may not be available for very fine spatial grains. At least one recent effort has addressed such limitations, discussing data collection protocols and gathering trait datasets that include data on traits by species, as well as ecological communities, geographic location, and environmental data by site (Jeliazkov et al., 2020). We advocate for extending this approach to include trait data on individuals per site (Figure 3) so that metrics of intraspecies trait variation relevant to urban eco‐evolutionary studies can be assessed (Alberti et al., 2017). We also recognize the difficulties of obtaining environmental data due to temporal, material, and economic constraints. Thus, we advocate for data collectors to provide data on measured traits by individuals and sites with species and geographic indicators, so that either they or their collaborators can obtain values for relevant environmental characteristics from the available sources. In this toolkit, we will provide:

1. *An online hub for researchers to find databases with global urban‐associated traits*: While still a burgeoning sub‐discipline, researchers have begun to collect data about the traits of urban species across urban habitat mosaics and relative to traits of the same species in surrounding wildlands. We will provide an online list of freely available trait data, along with any data about abiotic conditions that are available. This is a tool that will grow as users of the website share their data (with proper acknowledgment) and provide information about other datasets of which they are aware. Also, see the experiment toolkit.
2. *An online hub for researchers to find databases with trait data that are not urban‐specific*: There is a wealth of freely available trait databases for organisms living outside of cities. We will provide a repository for these datasets, as they will likely include data that provide important context for urban comparative studies. This is a tool that could also grow into a space for considering other kinds of trait‐based knowledge.
3. *Protocols and tips for collecting trait and environmental data across broad geographic scales*: One of the most powerful aspects of global trait mapping is that scientists can compare traits across global cities and contexts. Therefore, it will be useful to have a space for researchers to share their trait‐collecting methods and tips, as well as any lessons learned after collecting, archiving, and assessing trait data. We will provide this space as a third tool in the trait mapping toolkit. It will be another tool that has the potential to grow as users become co‐creators of content (with proper acknowledgment).

### 3.4 Common methods and freely available datasets for cross‐city manipulative experiments and meta‐analyses

In the age of digital and online databases, cross‐city collaborative research can be globally facilitated through the public sharing of resources and the creative use of online materials collected for initially very different purposes. As emerging technologies have become more affordable, techniques have come with ever‐increasing expectations of larger datasets, more samples, and populations, which ironically means these more affordable applications have become even less accessible to many. Making these emerging techniques and data collection more accessible will increase the diversity in the types of questions that can be asked, and make strides toward removing biases and misconceptions in urban evolutionary ecology research (Winchell et al., 2022). In the following toolkit, we identify online initiatives, networks, databases, and strategies for cross‐city experiments to create an inclusive environment where all scientists feel they can contribute to and have access to this research.

Cross‐city experiments include online databases, techniques, and tools that enable urban data to be comparatively interrogated and analyzed across taxa and communities in other ecosystems. Raw data repositories such as Dryad and DataOne are more than simply data storage, but instead include methods, samples, and analyses, which provide curated and standardized metadata for larger pattern analyses. For example, Miles et al. (2019) used these data to access metadata from 167 global urban studies to show evidence of urban‐facilitated gene flow in 33% of the studies, and Schmidt and Garroway (2022) reanalyzed 39 terrestrial vertebrate species datasets across 268 U.S. cities to find evidence of systemic racism leading to reduced urban connectivity; both studies identified patterns not seen in the individual published studies alone. As these databases grow, networks such as the UrbBioNet and Nutrient Network have emerged with foci on standardizing data collection, ethical behavior, and research questions, and that openly invite people to join from anywhere globally to share data and publish collaboratively (Aronson et al., 2017; Hautier et al., 2015). With the advent of remote‐sensing technologies, there are also many online sites that make available high‐resolution mapping data on light and air pollution, land cover and ecosystem characteristics, surface temperature data, and imperviousness, just to name a few, which enable comparative analyses between urban and non‐urban areas across the globe in association with biotic or abiotic factors (Zhao et al., 2022).

There are also a growing number of social media and community science sites, such as iNaturalist and eBird, where pictures and videos collected by anyone can provide vast amounts of data, much of which was not collected with the intent of addressing ecology and evolutionary biology questions. We point to several initiatives, like iEcology (Jarić et al., 2020; see Box 1), that have originated with the intent of building a more inclusive and practical framework, by which to conduct global research using these online media databases and that could easily be applied to urban areas. We believe these combined efforts of collaborative database sharing and social media outreach will prove to be the mitigating tools in increasing the contribution of underrepresented countries and institutions to cross‐city experiments that are necessary to our understanding of the global impact of urbanization on eco‐evo dynamics.

BOX 1 **iEcology as a model online initiative for facilitating cross‐city experiments**

**FIGURE B1:**
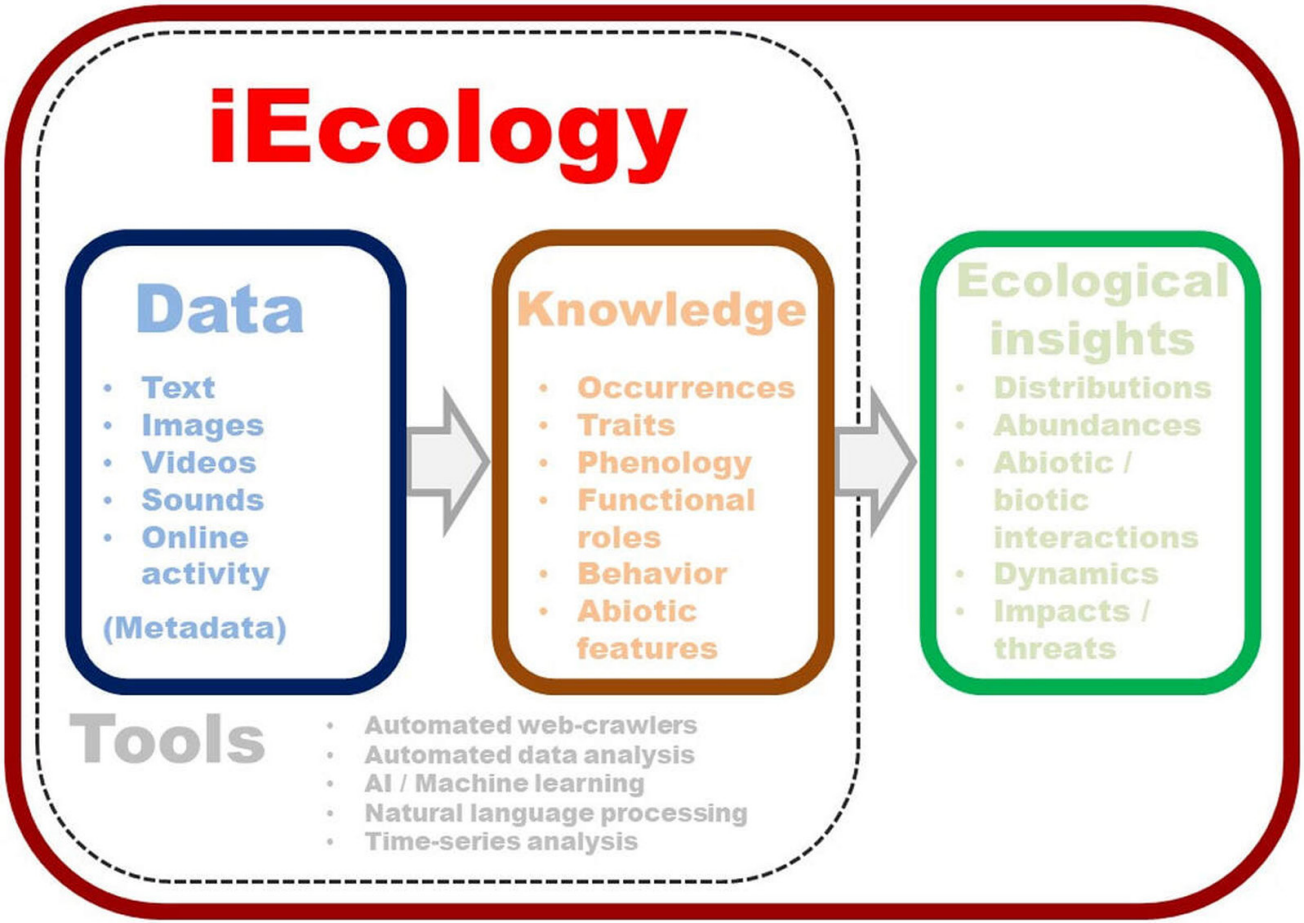
Schematic describing how the iEcology platform works. Reprinted with permission from Jarić et al. (2020).

The iEcology (internet ecology) initiative (Jarić et al., 2020)—born from the vast and ever‐increasing online data resources available to all – provides a framework for the highly collaborative use of digital resources. A key point is that unlike other large databases, this framework takes advantage of many social media outlets where the data were not initially collected for ecological analyses. As previously noted, one of the challenges of cross‐city experiments is that many individuals use different methods and technologies for collecting data, yet the iEcology approach embraces these unavoidable diverse characteristics. One method is to use social media platforms such as Flickr, news articles, Twitter, YouTube, Facebook, and Google Trends that collect data unknowingly in nature on species distributions, behaviors, and interactions. For example, Jagiello et al. (2019) used data collected via YouTube videos that showed behavioral differences between urban and rural red (*Sciurus vulgaris*) and gray (*Sciurus carolinensis*) squirrels across Europe. Møller and Xia (2020) observed video recordings of feeding from the human hand by 36 bird species across Europe to show this behavior is linked to urban habitat. iEcology also welcomes opportunities for community science involvement (Ellwood et al., 2023), making ecological research more inclusive and accessible to the public. Arazmi et al. (2022) used the community science database, eBird, to estimate distribution and occurrence of the invasive species Javan myna (*Acridotheres javanicus*), showing that it is highly invasive in Malaysia and its abundance is linked to urban area expansion. Jarić et al. (2020) identified future technologies that, through development, would further enhance the framework of iEcology that focuses on tools and online resources, including apps and games, automated content analysis (i.e., automatically identifies content), bioacoustics and ecoacoustics recording and interpretation, blockchain and linked data list development, Internet of Things (i.e., the network of computers and the online community), open source hardware for data collection and storage, and web scraping or the automatic retrieval of online content as data mining (Figure B1). Importantly, although the iEcology has mostly been applied to ecological systems, it has the potential to advance cross‐city experiments in evolutionary ecology that fosters equitable collaborations.

Looking to the future, one of the main challenges associated with cross‐city experiments remains how to standardize data collection, analyses, and even interpretation. Field and lab work is time‐ and personnel‐intensive and expensive, with added difficulties across global cities, especially for scientists in the Global South (Shackleton et al., 2021). We developed the following toolkit to facilitate comparative experiments and meta‐analyses across different cities that are global in scope yet led by grassroots efforts of local researchers.

1. *Existing international research networks and initiatives*: We will compile lists of existing research networks and initiatives that individuals interested in urban evolutionary ecology can join. These networks can also inspire the development of new collaborative global research.
2. *Data resources for comparative research*: As more data‐rich tools become available to researchers studying urban evolutionary ecology, we need to work with data scientists to assess freely available data that were collected from studies with diverse methods over broad spatiotemporal scales (Jarić et al., 2020; Kulkarni & Di Minin, 2021). To facilitate this work, we compiled freely available datasets that will facilitate cross‐city and meta‐analytic research, including raw data depositories and remote sensing and mapping databases.
3. *Tools for planning new cross‐city experiments*: Truly standardizing all research endeavors in urban evolutionary ecology will not only stymie creativity but also has the potential to exacerbate existing disparities in science. Instead, we provide common resources that can help international teams to plan their own initiatives, using an à la carte‐style approach. We include international funding opportunities and tips for collecting, analyzing, and curating data using standard practices. For example, online resources like CoordinateCleaner (Zizka et al., 2019), Biodiversity Data Cleaning (Ribeiro et al., 2022), and GloBI (Poelen et al., 2014), use software to automatically track datasets online, and can clean and correct taxonomic, spatial, and temporal errors for cross‐city analyses. This tool will grow as these initiatives grow, and our hope is that we will be able to include diverse ways of knowing as part of the tool in the future. Similarly, while we point to a few examples of agencies that financially support international research, like the United Nations, we recognize that funds are typically dispersed domestically, and we call for governments to work together to establish online collaborative international funding agencies.

### 3.5 Best practices and freely available resources for public outreach and communication of research findings in urban evolutionary ecology

Effective communication of evolutionary ecology research is especially important in urban systems due to their inherent connection and proximity to the public. Indeed, cities are becoming the spaces where people have their most frequent interactions with nature, molding their understanding and perception of the natural world. As natural areas are transformed by anthropogenic activities, it will also become more important that scientific research on urban organisms, evolution, and ecosystems reaches the target audiences, such as urban planners and decision makers, that can use those data to create implementable actions for sustainable development and conservation.

What effective communication of scientific research looks like depends, in part, on the goals, the intended audience, and the spatial scale (i.e., local or global). Outreach can be active or passive. For example, sidewalk signs can educate the community about urban flora and fauna passively or local coffee shop lectures can engage the community more actively. It can also take several forms: from scientific articles to popular literature, presentations to videos or documentaries, public workshops to community projects.

The communities in which we work have a vested interest in urban outcomes, including scientific research on urban ecology and evolution. As members of local communities, urban researchers often work in close contact with nonscientists, which offers opportunities for this essential community outreach and translation of our science. For example, researchers have designed letters to the community with brief information about their research and come to field sites prepared to answer questions from curious community onlookers (see Dyson et al., 2019). Similarly, some urban researchers distribute stickers to community members while conducting research that features the organisms being studied as a means of introducing the research and the wildlife. Scaling up to a more global audience, there have been a few documentaries that highlight urban evolution. For example, the BBC's Planet Earth series included the urban ecosystem as one of the focus systems and the Smithsonian documentaries “Laws of the Lizard” and “Miami Wild” which featured urban ecology and evolution research.

Another effective mode of scientific communication is connecting with educators to translate research into learning opportunities. Platforms that connect scientists and educators, such as Skype a Scientist (https://www.skypeascientist.com/), are excellent resources for outreach. Successful and long‐term partnerships have been forged between scientists and educators, some of which have resulted in the development of a curriculum focused on urban evolutionary ecology (Box 2). In general, the curriculum should be appropriate for the grade level and align with national educational standards. Since scientists do not always know the details of how an educational program can best align to these standards, it is best to collaborate with teachers and co‐design a curriculum that is appropriate and engaging for students.

BOX 2 **Urban Sci‐comm initiatives**

Here we highlight two scientific communication initiatives that currently exist as resources for disseminating urban evolutionary ecology research to different audiences.

**Scientist to scientist and community: Life in the City**

**FIGURE B2:**
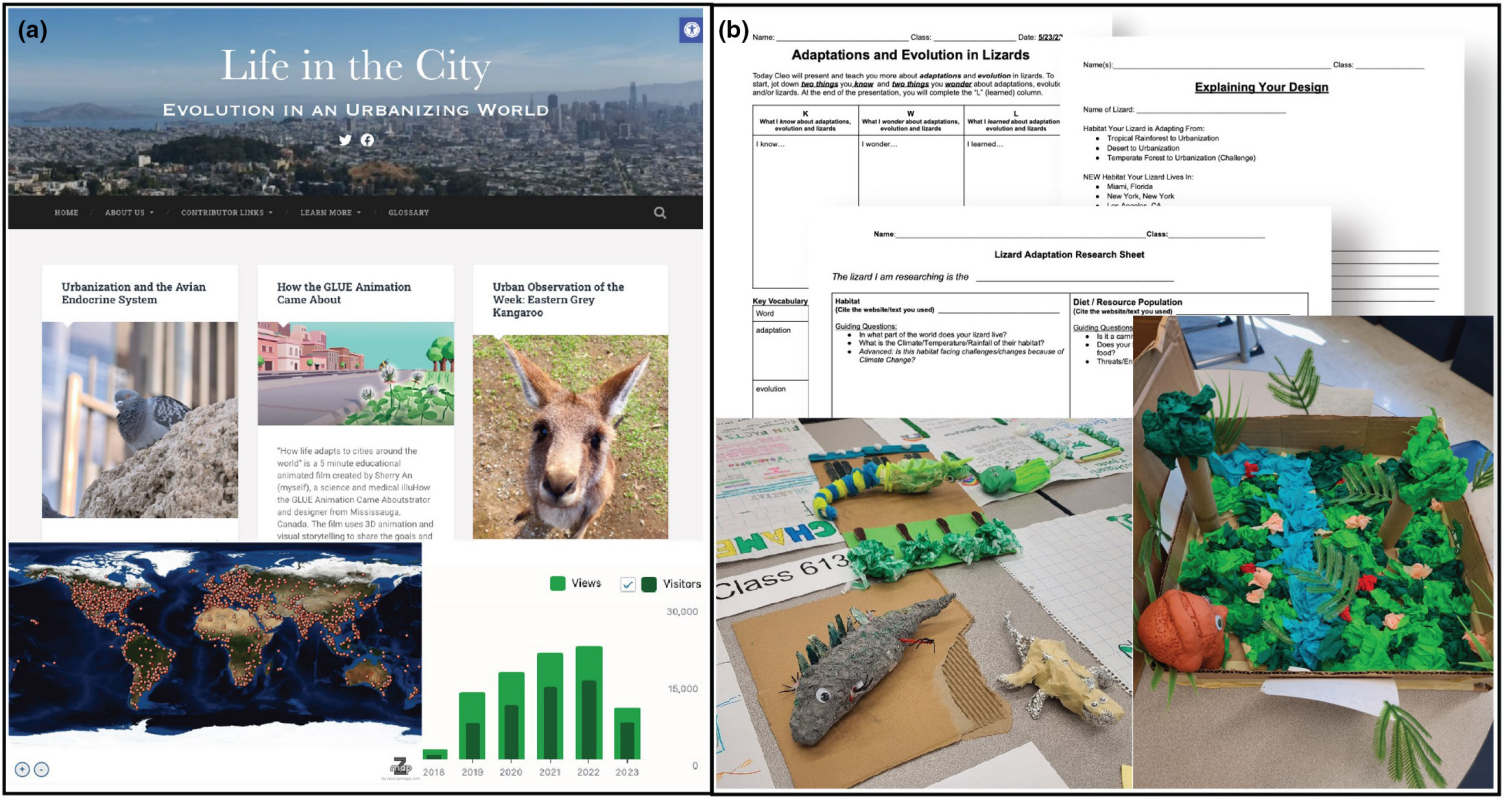
(a) “Life in the City” is an urban evolutionary ecology‐themed blog with a scientific and non‐scientific audience. Thousands of visitors from all over the world have engaged with the blog since it was created in 2018 (world map of visits by RevolverMaps.com). (b) The “Lizard Adaptation Challenge” is a successfully implemented educational activity that challenges students to think about ecology and evolution in urban environments and then build models of lizards based on their research and ideas.

Launched in 2018 by Elizabeth Carlen, Lindsay Miles, and Kristin Winchell, Life in the City (https://urbanevolution‐litc.com/) became the first urban evolutionary ecology blog (Figure B2a). Their vision was to unite the urban research community and simultaneously engage the public and scientists alike. The blog is free and publicly available and was intended to create an online community where research and ideas could be openly shared and discussed. The language is written in a way that is accessible to the public, with little scientific jargon included and a glossary with scientific terms defined.

The initial launching of the blog required a concerted effort by the founding editors to co‐recruit contributors, reach the intended readership through existing social media networks (primarily Twitter, on which the founding editors had a large following), and develop the base content so that posts would be regular and engaging for the first several months. This strategy brought in many readers and contributors: the site grew to include 36 posts by 13 contributors with more than 3000 views within the first four months. To reach a broader audience, the co‐editors also promote the blog at scientific conferences, invited seminars, and workshops, giving out Life in the City stickers and recruiting new collaborators.

Regular posts on Life in the City span a range of content types meant to engage different audiences. For example, one post category, the “urban observation of the week” is a weekly featured urban organisms found in the “wild” to highlight the incredible biodiversity that exists in cities but that often goes unnoticed. Another post category, “research summaries,” provides a short explanation of a recent scientific article, explaining what was done and how, and why the results matter. These research summaries have received praise from educators and students at both K‐12 & university levels as a means to make scientific articles more accessible and understandable. The third main type of post is a simple “new literature alert” which shares a recent article abstract and link to the publication, with the main goal of helping the scientific community stay up‐to‐date on relevant literature. Often researchers who are featured in this type of post become contributors to the blog and write a research summary post.

Life in the City was intended to be an open and inclusive community from the start. The blog editors invite contributors from around the world and share posts on Twitter and Facebook. Contributors include community members (e.g., students from The Bronx High School of Science), undergraduate and graduate students, and both early career and senior researchers. With more than 450 posts written by 92 contributors as of early 2023, Life in the City has reached a global audience, regularly receiving >100 visitors a day. The blog continues to grow and keeps inclusivity at its core, adding new editors, content types, and collaborative efforts with educators. Key challenges for the future will be to diversify content to reach different types of audiences (e.g., video integration) and expand the reach to include non‐english language researchers and audiences. An ongoing challenge has been to identify funding sources to maintain the blog, and with plans of expanding future content, additional funding sources will need to be identified.

**Scientist to educators & community: Lizard adaptation challenge**

As part of Skype‐A‐Scientist, Ph.D. student Cleo Falvey connected with middle school educators, Jacalyn Fiechter and Maire McCormack. The interaction was so rewarding for everyone involved, that in the following months, they worked together to develop the “Lizard Adaptation Challenge” to teach students about adaptive morphological changes in urban environments. Specifically, students learn about adaptations in lizards, how they relate to habitat use, and the morphological changes that have been documented in urban *Anolis* lizards (e.g., Falvey et al., 2020; Winchell et al., 2016). Students then research their own chosen species of lizard and think about what adaptations that lizard might have if the lizard were to live in the city, as well as any trade‐offs the lizard might need to make to live in its new urbanized habitat. The project culminates in an engaging creative project that involves the construction of a physical lizard model made from recycled materials, as well as a presentation to their class and wider community (Figure B2b).

This educational activity has been delivered to over 200 middle schoolers in a public school in New York City, with a student population largely comprised of students from historically excluded groups in STEM fields. In addition, students who were not directly involved in the project were still able to participate by listening to the presentations and doing “gallery walks” around the room with the models, so more than 500 students had the opportunity to learn about adaptations and urban evolutionary ecology.

Although this project was designed specific to lizard adaptations and created directly with the teachers who were going to implement the curriculum, it could be scalable to any urban species and any grade level. In addition, since the lizards are constructed using recycled materials that the students collect themselves, the costs for running the interactive part of the activity are low. Currently, efforts are underway to publish the curriculum as an open‐source activity that can be disseminated on the internet to teachers around the world.

Although there are many traditional ways in which scientists can engage with other scientists, including textbooks, journal articles, and scientific conferences, the reach of these products can be extended significantly through scientific communication initiatives. For example, many of these resources, including books and journal articles, have been summarized in the “Life In the City – Urban Evolution” blog (https://urbanevolution‐litc.com/) as well as promoted on Twitter (see Box 2). In this toolkit, we provide tools to facilitate effective scientific communication of research in urban evolutionary ecology. Specifically, we will identify existing urban evolutionary ecology science communication initiatives, compile science communication resources that could be adapted for urban evolutionary ecology research, and provide ideas for new initiatives.

1. *Connecting urban evolutionary ecology researchers with scientific communicators*: There are many talented artists, filmmakers, writers, and other scientific communicators who could help researchers communicate their findings to public audiences. We incorporated a Google form into the MURP (Appendix S3) to specifically identify scientific communicators who are interested in working with evolutionary ecologists globally. We also compiled a list of freely available documentaries, sci‐comm articles, and blogs so that researchers can see possible avenues for communicating their own science.
2. *Practical tools for formal and informal scientific communication*: Although a handful of these tools exist for urban evolutionary ecology scientific communication, there are many additional outreach tools already available but not yet specific to this field of research. One avenue to build up the urban eco‐evo toolbox is to build on current scicomm tools such as Sketchfab 3D models (https://sketchfab.com/) and the Art+Bio Collaborative (https://www.artbiocollaborative.com/), among others. Since the infrastructure and audience is already established for these tools, modifying them, either via spin‐off projects or collaborative efforts, to specifically focus on urban ecology and evolution could be easier and more fruitful than building new resources from scratch. We will also include tools and examples of successful integration of ecology and evolution in classrooms (e.g., https://robdunnlab.com/teaching‐resources/).
3. *Ideas for future initiatives and freely available outreach materials*: In addition to co‐opting current sci‐comm tools, the outreach toolkit plans to generate new initiatives. To advance research with communities, we will generate infocards, booklets, and stickers that are broadly related to urban eco‐evo research and can be handed out to community members while conducting fieldwork. For a more passive approach, we plan to include “print‐at home” materials such as signs that explain urban biodiversity and coloring books. Templates and examples of these resources will be featured on the website for researchers and community members to download and use free of charge. We will also work on implementing translated materials, with the option of users submitting their own translated versions of these materials using a Google form (Appendix S4).

## 4 CONCLUSIONS

Integrating multidisciplinary and diverse teams in urban evolutionary ecology requires optimizing and developing tools and skills that integrate the scientific community and facilitate international collaborations. Here, we aim to promote a more inclusive, equitable, and collaborative approach to research in urban evolutionary ecology by providing an online collaborative research hub for toolkits that facilitate the equitable establishment of international, collaborative teams of researchers, help researchers find available data, and other resources for designing new studies, and assist international researchers in communicating their research to public audiences and other scientists. Each toolkit is user‐friendly, includes freely available tools, and has mechanisms for growing as users become fully acknowledged co‐creators of website content. We recognize that each urban community is unique and particular strategies for decolonizing research in each urban system will look different. Nevertheless, we hope our toolkits will help others get started in decolonizing their urban evolutionary ecology research, forming international, collaborative research partnerships, conducting equitable international evolutionary ecology research, and effectively communicating their findings. After assembling these toolkits, we recognize and respectfully acknowledge that much of this information is already being applied to other ecosystems. Thus, we must be creative in thinking about how online resources can be repurposed and adapted for urban ecosystems, a practice alone that will make global collaborations more inclusive. Overall, although the use of these tools does not directly guarantee equity and cultural sensitivity in collaborations, the use of these and other associated collaborative tools has the potential to allow alternative perspectives and points of view to be expressed and potentially taken into account while conducting research. Finally, we recognize that many of the authors of this article work in the Global North (although some of the authors are from the Global South). While we support the assertion that much of the work of creating more equitable systems should be borne by scholars from regions that have historically benefited from colonial systems of power, we also welcome further discussion as we co‐create these toolkits with users and are particularly interested in building greater capacity in terms of diversity and intersectionality.

## AUTHOR CONTRIBUTIONS

**Amy M. Savage:** Conceptualization (lead); funding acquisition (lead); methodology (lead); writing – original draft (lead); writing – review and editing (lead). **Meredith J. Willmott:** Conceptualization (supporting); methodology (equal); project administration (lead); writing – original draft (supporting); writing – review and editing (supporting). **Pablo Moreno‐García:** Writing – original draft (supporting). **Zuzanna Jagiello:** Conceptualization (equal); visualization (equal); writing – original draft (equal). **Daijiang Li:** Writing – original draft (supporting). **Anna Malesis:** Writing – original draft (supporting). **Lindsay S. Miles:** Conceptualization (supporting); writing – original draft (equal). **Cristian Román‐Palacios:** Writing – original draft (supporting). **David Salazar‐Valenzuela:** Conceptualization (supporting); writing – original draft (supporting). **Brian C. Verrelli:** Conceptualization (equal); methodology (equal); writing – original draft (equal). **Kristin M. Winchell:** Methodology (supporting); writing – original draft (supporting). **Marina Alberti:** Conceptualization (equal); writing – original draft (equal). **Santiago Bonilla‐Bedoya:** Writing – original draft (equal). **Elizabeth Carlen:** Conceptualization (supporting); writing – original draft (supporting). **Cleo Falvey:** Writing – original draft (supporting). **Lauren Johnson:** Conceptualization (supporting); writing – original draft (supporting). **Ella Martin:** Writing – original draft (supporting). **Hanna Kuzyo:** Writing – original draft (supporting). **John Marzluff:** Conceptualization (supporting); writing – original draft (supporting). **Jason Munshi‐South:** Conceptualization (supporting); writing – original draft (supporting). **Megan Phifer‐Rixey:** Conceptualization (equal); writing – original draft (equal). **Ignacy Stadnicki:** Conceptualization (equal). **Marta Szulkin:** Conceptualization (equal); writing – original draft (supporting). **Yuyu Zhou:** Conceptualization (equal). **Kiyoko M. Gotanda:** Conceptualization (lead); methodology (lead); project administration (equal); writing – original draft (lead); writing – review and editing (lead).

## CONFLICT OF INTEREST STATEMENT

The authors of this article do not have any conflicts of interest to report with respect to this manuscript.

## Supporting information


Appendix S1

Appendix S2

Appendix S3

Appendix S4

Data S1

## Data Availability

Data sharing is not applicable to this article as no datasets were generated or analyzed during the current study.
