# Supplementary material for: Online toolkits for collaborative and inclusive global research in urban evolutionary ecology
Source: Ecol Evol. 2024 Jun 25;14(6):e11633. doi: 10.1002/ece3.11633 (PMC11197044; doi:10.1002/ece3.11633)
Supplement: Supplementary file 1 — Appendix S1 [file ECE3-14-e11633-s001.pdf]

Urban Researcher Form

Please fill out this form if you are working in the fields of urban ecology or evolution, or are producing work affiliated with those fields. This form will add your location and information on a map that will act as a database and resource for those in the field of urban ecology and evolution.

* Indicates required question

1. Email *

____

2. What is your name? *

____

3. What is your current position?

Mark only one oval.

- ◯ Undergraduate Student
- ◯ Graduate Student
- ◯ Postdoc
- ◯ Professor
- ◯ Lab Technician
- ◯ Industry-Affiliated Researcher
- ◯ Government-Affiliated Researcher

4. What is your focus within the field of Urban Ecology and Evolution? *

Check all that apply.

- [ ] Aquatic Ecology
- [ ] Plant Ecology
- [ ] Applied Ecology
- [ ] Animal Ecology
- [ ] Habitat Ecology
- [ ] Sociology
- [ ] Biogeography
- [ ] Environmental Engineering
- [ ] Evolutionary Ecology
- [ ] Behavioral Ecology
- [ ] Taxonomy
- [ ] Evolutionary Biology
- [ ] Computational Biology
- [ ] Geography

5. What is your research focus (please fill this out if you would like to elaborate on the multiple choice).

____

6. Is your research affiliated with an LTER (Long Term Ecological Research Network)? If so, please name the LTER, if not, leave blank.

____

7. What is your latitude where your institution is located? Decimals please! *
Example: 39.2394

____

8. What is your longitude where your institution is located? Decimals please! *

   Example: -75.2409

   ____

9. What is your website or lab website? If you do not have one, please provide an email that can be used to reach you. *

   ____

This content is neither created nor endorsed by Google.

Google Forms
