# Supplementary material for: Online toolkits for collaborative and inclusive global research in urban evolutionary ecology
Source: Ecol Evol. 2024 Jun 25;14(6):e11633. doi: 10.1002/ece3.11633 (PMC11197044; doi:10.1002/ece3.11633)
Supplement: Supplementary file 2 — Appendix S2 [file ECE3-14-e11633-s005.pdf]

Urban Ecology Project Form

Hello! This form collects data from ongoing projects in Urban Ecology; please fill it out with your information. If your project has multiple study sites, please fill the form out multiple times, and just change the latitude and longitude for each study site.

* Indicates required question

1. What is the name of your project? *

2. What is your name? *

3. Are you affiliated with a University or Research Institute? Please let us know which one!

4. What best describes your area of study?

   Mark only one oval.

   - Ecology
   - Evolution
   - Environmental Engineering
   - Sociology
   - Bioinformatics

5. Is this project affiliated with an LTER (Long Term Ecological Research Network)? If yes, please name which one, if not, leave blank.

6. What is the latitude of your study area? **Please respond in decimal degree**. If your study spans multiple locations please choose one. *

   ex: 39.9526

7. What is the longitude of your study area? **Please respond in decimal degree**. If your study spans multiple locations please choose one. *

   ex: 39.9526

8. Because many ecological studies span a variety of locations, please use this question to list additional study areas if you have used them- you do not need to add latitude and longitude, just the city, state (if appropriate), and country that the research is located in:

   ex: "Albuquerque, New Mexico, United States"

9. What is the status of your project? *

Mark only one oval.

- Completed with a publication
- Ongoing
- Not Applicable

10. Is your study area in an urban, non-urban, or mixed site? *

Mark only one oval.

- urban
- non-urban
- mixed

11. Please provide a brief description of your research project. 1200 character limit *

____

12. Please provide a link to your project's website or a site with further information. *

____
____
____
____
____

This content is neither created nor endorsed by Google.

Google Forms
