# Supplementary material for: Online toolkits for collaborative and inclusive global research in urban evolutionary ecology
Source: Ecol Evol. 2024 Jun 25;14(6):e11633. doi: 10.1002/ece3.11633 (PMC11197044; doi:10.1002/ece3.11633)
Supplement: Supplementary file 3 — Appendix S3 [file ECE3-14-e11633-s004.pdf]

Science Communicator Form

This form is for the global use of science communicators to network and find collaborative opportunities with eco-evo researchers who are carrying out projects in their communities and geographic regions.

* Indicates required question

1. What is your name? *

2. What medium do you work in? *

3. Are you affiliated with any scientific institutions (museums, journals, societies, etc)? If yes, please provide the name of the institution(s), if no, please leave blank

4. What is your website?

5. What is your social media account?

6. Please provide an email you are comfortable being accessed for collaboration.

7. What is the latitude of your city? **Please respond in decimal degree**. *

ex: 39.9526

8. What is the longitude of your city? **Please respond in decimal degree**. *
ex: 39.9526

This content is neither created nor endorsed by Google.

Google Forms
