# Supplementary material for: Online toolkits for collaborative and inclusive global research in urban evolutionary ecology
Source: Ecol Evol. 2024 Jun 25;14(6):e11633. doi: 10.1002/ece3.11633 (PMC11197044; doi:10.1002/ece3.11633)
Supplement: Supplementary file 4 — Appendix S4 [file ECE3-14-e11633-s002.pdf]

*

Collaborator Form

If you have a suggestion for one of these tools in the toolkit please fill this out!

* Indicates required question

1. Which tool are you addressing? *

Mark only one oval.

- Toolkit 1: Name
- Toolkit 2: Name
- Toolkit 3: Name
- Toolkit 4: Name
- Toolkit 5: Name

2. What is your suggestion? *

____

3. Would you like to be credited for this idea? *

Mark only one oval.

- Yes
- No

4. What is your name? *

____

5. What is an appropriate email to contact you with? *

____

This content is neither created nor endorsed by Google.

Google Forms
