# Supplementary material for: Online toolkits for collaborative and inclusive global research in urban evolutionary ecology
Source: Ecol Evol. 2024 Jun 25;14(6):e11633. doi: 10.1002/ece3.11633 (PMC11197044; doi:10.1002/ece3.11633)
Supplement: Supplementary file 5 — Data S1 [file ECE3-14-e11633-s003.docx]

**Supplementary information:**

Supplemental document 1: Google Form for researchers in the field of urban evolutionary ecology

Supplemental document 2: Google form for urban evolutionary ecology projects

Supplemental document 3: Google form for science communicators interested in working with urban evolutionary ecology researchers

Supplementary document 4: Toolkit contribution form
